# Distribution and Abundance of Parasites of the Rhodesgrass Mealybug, *Antonina graminis*: Reassessment of a Classic Example of Biological Control in the Southeastern United States

**DOI:** 10.1673/031.009.4801

**Published:** 2009-07-02

**Authors:** Jillian M. Chantos, S. Bradleigh Vinson, Ken R. Helms

**Affiliations:** ^1^ Department of Entomology, Texas A&M University, College Station, TX 77843–2475; ^2^ Department of Biology, University of Vermont, Burlington, Vermont 05405

**Keywords:** Neodusmetia sangwani, mealybug, parasitoid

## Abstract

Control of the rhodesgrass mealybug, *Antonina graminis* Maskell (Hemiptera: Pseudococcidae), by the encyrtid wasp *Neodusmetia sangwani* is considered a textbook example of classical biological control. However, recent evidence suggests that *A. graminis* is abundant in the southeastern United States and no recent surveys have been conducted to determine the status of *N. sangwani* or other *A. graminis* parasites. A survey was conducted and it was found that *N. sangwani* was uncommon overall, occurring at only 20 percent of survey sites. In addition, *N. sangwani* exhibited a patchy geographic distribution. Possible causes for these results are that *N. sangwani* has not dispersed widely since its introduction, or that the imported fire ant, *Solenopsis invicta*, is interfering with biological control. These results suggest that a reevaluation of the efficacy of biological control may be necessary. The survey also found two other encyrtid wasps utilizing *A. graminis* as a host. One, *Acerophagus* sp., is apparently native and was nearly as frequent as *N. sangwani*, while the other, *Pseudectroma* sp., is apparently introduced and relatively rare.

## Introduction

The approach of classical biological control is to reduce the negative effects of pest organisms by introducing their parasites, predators, or pathogens. When successful, natural enemies become established and reduce the pest's frequency, while its continued presence at low frequency maintains enemy populations, resulting in self-sustaining, long-term control (Stern and van den Bosch 1959; Debach 1964). Previously successful classical biological control programs were directed at pest insects, for example, the stink bug, *Nezara viridula* (Davis 1967), the olive scale, *Parlatoria oleae* (Huffaker and Kennett 1966), the walnut aphid, *Chromaphis juglandicola* (van den Bosch et al. 1970), and the carrot aphid, *Cavariella aegopodii* (Stern and van den Bosch 1959).

An important and widely cited example of the success of classical biological control is that of the rhodesgrass mealybug, *Antonina graminis* Maskell (Hemiptera: Pseudococcidae), in the southeastern United States (Dean et al. 1979; Caltagirone 1981). Described in 1897 from specimens discovered in Hong Kong, China, *A. graminis* is believed to be of Asian origin, however it is invasive and now occurs nearly worldwide in tropical and subtropical regions (Dean et al. 1979; Ben-Dov et al. 2006a). It feeds on grasses, and exhibits a tremendous host range with records from well over 100 grass species in over 50 genera, which is likely to facilitate its successful invasion (Ben-Dov et al. 2006b). Currently, over 70 species of grasses are reported as hosts for the southeastern United States alone (Chada and Wood 1960; Helms and Vinson 2000). In addition, *A. graminis* reproduces parthenogenetically, and it is inconspicuous, occurring on the crown and under leaf sheaths of the plant (Chada and Wood 1960).

When first discovered in the United States in 1942, *A. graminis* was already a serious pest of rangeland grasses in south Texas (Chada and Wood 1960), and soon afterwards in Florida (Questel and Genung 1957). Biological control programs in these States implemented the introduction of five encyrtid wasps: *Anagyrus antoninae* from Hawaii, *Pseudectroma europaea* (= *Timberlakia europaea*), *Xanthoencyrtus phragmitis*, and *Boucekiella antoninae* from France, and *Neodusmetia sangwani*, from India. *Anagyrus antoninae* was introduced into Texas in 1949 and Florida in 1954, *P. europaea* was introduced into Texas in 1954–1955, and Florida in 1959, *X. phragmitis* and *B. antoninae* were introduced into Texas in 1954–1955, and *N. sangwani* was introduced into Florida in 1957, and Texas in 1959 (Riherd 1950; Questel and Genung 1957; Dean and Schuster 1958; Schuster et al. 1971; Schuster and Dean 1976; Bennett 1993).

Introductions of *A. antoninae, P. europaea, X. phragmitis*, and *B. antoninae* were unsuccessful. While established initially, *A. antoninae* was apparently unable to withstand high summer temperatures and/or compete effectively with *N. sangwani*, only occurring at low frequency in the last reported Texas survey in 1964–65, while it was not found in Florida surveys from 1975–1991 (Schuster and Dean 1976; Bennett 1993). Neither *X. phragmitis, P. europaea*, or *B. antoninae* were detected following their introduction and presumably did not become established (Schuster et al. 1971; Bennett 1993). The introduction of *N. sangwani* was highly successful, however, and it was reported to reduce *A. graminis* populations by 68.8% in Texas, and in the Rio Grande Valley by 50 to 83% (Schuster and Dean 1976). The introduction of *N. sangwani* was also successful in Florida; surveys from 1975–1991, indicated that *N. sangwani* was abundant throughout this period (Bennett 1993). The successful control *A. graminis* with *N. sangwani* has also now been reported in Brazil and Israel (Machado da Costa et al. 1970; Gerson et al. 1975).

There is no information on the field status of the *A. graminis* biological control system during the past 15 years in Florida, and the past 40 years in Texas. New data are important because recent evidence suggests that *A. graminis* is widespread and abundant in the southeastern United States, and this species has the potential to severely impact agricultural and ecological systems (Helms and Vinson 2000, Helms and Vinson 2002). In addition, significant introductions of new alien species have occurred since earlier surveys, and these can interfere with existing biological control (e.g., Bartlett 1961; Reeve and Murdoch 1986; Reimer et al. 1993; Kaplan and Eubanks 2005). Importantly, *A. graminis* is now often associated in the southeastern United States with an invasive ant, the red imported fire ant, *Solenopsis invicta*, which could facilitate the abundance of *A. graminis* (Helms and Vinson 2002, Helms and Vinson 2003). Thus, we assessed the potential for continued control of *A. graminis* by conducting a survey of its current parasitoids, their distribution and abundance. Many successful cases of biological control are found in the literature, but long-term follow-ups on the frequency and efficacy of biological control agents are rare. Studies concerning the long-term establishment of biological control agents may provide beneficial information to maintaining pest suppression.

## Methods

**Figure 1.**
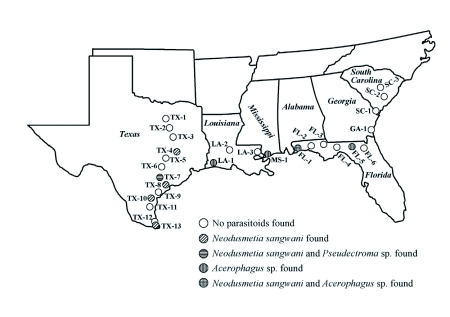
Occurrence of parasitoids that utilize *Antonina graminis* as a host found during our 2005 survey of the southeastern United States. Circles are centered over site locations.

Two surveys were carried-out to assess the abundance and distribution of parasitoids utilizing *A. graminis* as a host. Both surveys were conducted in unmanaged habitats dominated by grasses, including a substantial proportion of *A. graminis* host grasses. Burmudagrass (*Cynodon dactylon*) was targeted because it is an important preferred host plant of *A. graminis* in rangeland systems (Chada and Wood 1960). The first survey was conducted at 13 sites (TX1 – TX13) between Dallas and Brownsville, TX, in July, 2005, and was replicated three months later, in October. The months of our survey encompass the time of year when *A. graminis* population sizes are relatively large (Chada and Wood 1960). Sites were located along highways (Interstate 35, State Highway 6, and Highway 77) at 80 km intervals (Figure 1) with no more than a five m^2^ area sampled. Field estimates of a minimum of approximately 100 *A. graminis* were collected from *C. dactylon* at each site. *A. graminis* were clipped from plants, then counted, and placed into culture tubes with a water reservoir that provided a humid environment and prevented parasitoid escape. The tubes were housed in a rearing chamber at 30^°^C, and checked daily for parasitoid emergence for a minimum of 30 days, ensuring that any parasitoids present would have ample time to complete development (Schuster 1965).

The second survey was conducted without replication in October 2005, across the southeastern United States, east of Texas. Sites were located in Louisiana, Mississippi, Florida, Georgia, and South Carolina, and occurred at approximately 100–200 km intervals near highways (primarily Interstates 10 and 95). For most sites, field estimates of a minimum of approximately 100 *A. graminis* were collected from bermudagrass (*C. dactylon*) and/or crabgrass (*Digitana* sp.). After collecting, *A. graminis* were removed from plants, counted, and placed into a rearing chamber at 30°C as outlined for the Texas survey. Parasitoids that emerged from both surveys were counted, cleared, placed into 90% ethanol, and identified (Gibson et al. 1997). Parasitized *A. graminis* were identified by the presence of a parasitoid exit hole, and their numbers were counted.

## Results

In the Texas survey, parasitoids emerged from adult *A. graminis* at five (38.5%) of 13 sites, primarily in the southern half of the state (Table 1, Figure 1). At one site (TX–7), two species of parasitoids emerged, *N. sangwani* and *Pseudectroma* sp., while only *N. sangwani* emerged from *A. graminis* at the remaining four sites (Table 1, Figure 1). There was strong consistency across sampling periods in the Texas survey: where a parasitoid was found in July, it was normally found again at similar frequency at the same site in October. In addition, where parasitoids were not found in July, they were not found in October (Table 1). At Texas sites where parasitoids were found, the percent of *A. graminis* that were parasitized ranged from 1.6 to 15.0% in July and 2.8 to 13.1% in October (Table 1).

In the survey east of Texas, parasitoids emerged from *A. graminis* at four (25%) of 16 sites, two in Louisiana, and two in Florida. Two parasitoid species, *N. sangwani* and *Acerophagus* sp. emerged at one site in Florida (Fl–1), while only *Acerophagus* sp., emerged from the remaining three sites, two in Louisiana (LA–1, LA–4) and one site in Florida (FL–5) (Table 1, Figure 1). The percent of adult *A. graminis* that were parasitized at sites where they emerged ranged from 0.9 to 16.8 (Table 1). Pooling the results of both surveys within the southeastern United States, parasitoids were found at nine (31%) of 29 sites surveyed. Most common was *N. sangwani*, found at six sites, while *Acerophagus* sp. was found at four sites, and *Pseudectroma* sp. was found at a single site.

**Table 1.**
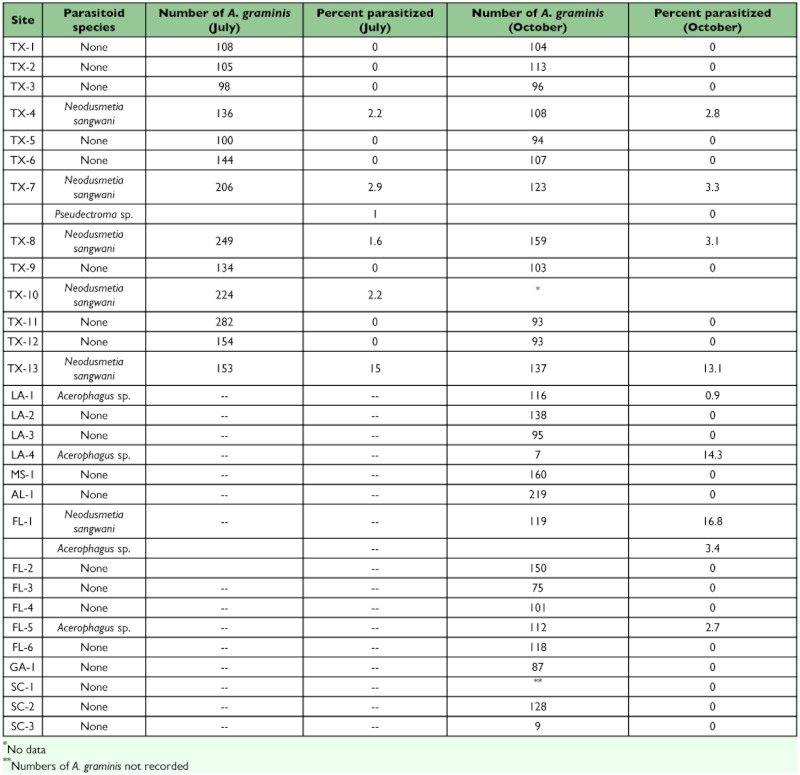
Parasitism of *Antonina graminis* at sites in Texas (TX) Louisiana (LA), Mississippi (MS), Alabama (AL), Florida (FL), Georgia (GA), and South Carolina (SC) in 2005.

## Discussion

While the control of *A. graminis* by *N. sangwani* is viewed as an important success of biological control, there is surprisingly little quantitative data on the frequency of either the host or parasitoid since shortly after initiation of the biological control program. In this study no quantitative data on the frequency of *A. graminis* is provided, however, it appeared common and often abundant at the study sites, which is consistent with reports from other recent studies in the southeastern United States (Helms and Vinson 2000, Helms and Vinson 2002). Given the frequency of *A. graminis*, it was surprising that *N. sangwani* was found at relatively few sites and that the rate of parasitism was generally low.

There are a number of possible, and non-exclusive, reasons why *N. sangwani* was uncommon. First, the introduction of the parasitoid was limited to specific geographic areas and *N. sangwani* may not have dispersed widely since its introduction. Females are flightless, and their ability to disperse naturally is estimated to be only 0.8 km/yr (Dean et al. 1979). Consistent with the possibility that the results may be influenced by limited dispersal, *N. sangwani* was introduced into southeast Texas (Schuster et al. 1971), and it was found primarily at sites in that region of Texas (Figure 1). Although the release sites for the parasitoid in Florida were unknown, *A. graminis* was only known at that time to be widespread in central to south Florida, and this is the region where *A. antoninae* was introduced (Questel and Genung 1957), suggesting that *N. sangwani* was introduced only into the southern half of the state. The Florida survey locations were, however, in the north, and *N. sangwani* was found at only a single site. In the other southeastern states, the parasitoid was apparently not introduced, and it was not found at sample sites within these states. These data are generally consistent with the role of limited natural dispersal in limiting the current range of *N. sangwani*, however, its potential importance would be overestimated if *N. sangwani* is commonly transported with *A. graminis* in commercial turf-grasses and similar products. Such human-mediated movements could, however, still result in patchy parasitoid distributions. If the distribution of *N. sangwani* results from limited dispersal abilities, then introducing the parasitoid into regions where it currently does not occur may prove valuable in limiting *A. graminis* populations.

A second possible reason why *N. sangwani* was unexpectedly uncommon is that the fire ant, *S. invicta*, is interfering with the biological control of *A. graminis*. In the southeastern United States, *S. invicta* is abundant and often associated with *A. graminis*, which it tends for honeydew and commonly encloses in shelters constructed from soil and debris (Helms and Vinson 2002). A recent study showed that the frequency of *A. graminis* increases with increasing proximity to colonies of *S. invicta*, which is consistent with *S. invicta* protecting *A. graminis* (Helms and Vinson 2003). Whether this is the case requires further research; however, recent evidence suggests that *S. invicta* does protect *A. graminis* from *N. sangwani* (Chantos, Vinson, and Helms, unpublished data).

*A. graminis* was parasitized nearly as frequently by a species of *Acerophagus* as it was by *N. sangwani*. Because *Acerophagus* is native to North America and the Caribbean (Gordh 1979), it is likely that it is a native species. Further, the frequency of *Acerophagus* sp. at survey sites was nearly equal to that of *N. sangwani* and is another indicator that *N. sangwani* is not providing widespread control of *A. graminis*. If so, the native parasitoid may be an important additional tool in the biological control of the mealybug.

While *Pseudectroma* sp. was found at only a single site, the discovery that it was a parasite of *A. graminis* is of interest. While *P. europaea* was one of the early parasitoids introduced, it was not believed to become established and has not been found in any subsequent survey. Indeed, it is likely that *Pseudectroma* sp. is not *P. europaea*. A *Pseudectroma* sp. identified as not being *P. europaea*, and presumably from Asia, was found utilizing *A. graminis* as a host in Florida surveys from 1975–1991 (Bennett 1993). It was common, although not as abundant as *N. sangwani* (Bennett 1993). The origin of this parasitoid and its potential impact on *A. graminis* is unclear but deserves further study.

The surveys involved a number of sites over a large range, and they suggest that the biological control of *A. graminis* from the parasitoid *N. sangwani* may not be as widespread or effective as when first released. This could occur because *N. sangwani* has failed to disperse widely, or because *A. graminis* is protected from parasitoids by *S. invicta*. Other causes are also possible. Further study would be valuable in exploring these causes and in determining the generality of our results. Because *A. graminis* is an important invasive species with a history of significant negative impacts on agriculture, it is important to determine whether its biological control remains effective or whether a new program may be warranted.
